# Investigating retroviral super-infection in wild chimpanzees (Pan troglodytes verus)

**DOI:** 10.1186/1742-4690-8-S1-A242

**Published:** 2011-06-06

**Authors:** Anja Blasse, Adeelia Goffe, Roger Mundry, Fabian H Leendertz, Sébastien Calvignac-Spencer

**Affiliations:** 1 Research group Emerging Zoonoses, Robert Koch-Institut, Berlin, Germany; 2 Max Planck-Institute for Evolutionary Anthropology, Leipzig, Germany

## Background

While the molecular epidemiology of retroviruses in wild primate populations has received much attention, the related question of the frequency and nature of super-infection events has remained largely neglected. Here, we explicitly investigated it, focusing on simian foamy viruses (SFV) infecting wild chimpanzees (Pan troglodytes verus), as an example.

## Methods

We first compared the costs and benefits of end-point dilution PCR (EPD-PCR) (currently considered a gold standard method) and multiple bulk PCR cloning, when applied to non-invasive samples. For the latter method, we had to develop a specific analytical framework aimed at deciphering relevant biological information from method-induced biases. We then applied multiple bulk PCR cloning to samples collected from chimpanzees belonging to different age classes.

## Results and discussion

We found that, when applied to faeces, proper EPD-PCR analysis will sometimes require the consumption of unrealistic large amounts of biological material (i.e. when “native” EPD-PCR conditions are encountered). However, super-infection status, as well as the underlying main strain sequences, could be robustly inferred from multiple bulk PCR analyses (which are more parsimonious), using a combination of statistic and network analyses. Applying this method to individuals belonging to different age classes, we finally found that wild chimpanzees actually accumulated distinct chimpanzee-specific SFV throughout their lives.

